# *Tg(Δ113p53:cmyc)* Transgene Upregulates *glut1* Expression to Promote Zebrafish Heart Regeneration

**DOI:** 10.3390/jcdd10060246

**Published:** 2023-06-04

**Authors:** Zimu Tang, Kaiyuan Wang, Lijian Lo, Jun Chen

**Affiliations:** 1 MOE Key Laboratory of Biosystems Homeostasis & Protection, College of Life Sciences, Cancer Center, Zhejiang University, Hangzhou 310058, China; 11807041@zju.edu.cn (Z.T.);; 2 College of Animal Sciences, Zhejiang University, Hangzhou 310058, China; g0403022@zju.edu.cn

**Keywords:** heart regeneration, glut1, cmyc, zebrafish, glucose transportation, cardiomyocyte proliferation

## Abstract

The heart switches its main metabolic substrate from glucose to fatty acids shortly after birth, which is one of reasons for the loss of heart regeneration capability in adult mammals. On the contrary, metabolic shifts from oxidative phosphorylation to glucose metabolism promote cardiomyocyte (CM) proliferation after heart injury. However, how glucose transportation in CMs is regulated during heart regeneration is still not fully understood. In this report, we found that the expression of Glut1 (*slc2a1*) was upregulated around the injury site of zebrafish heart, accompanied by an increase in glucose uptake at the injury area. Knockout of *slc2a1a* impaired zebrafish heart regeneration. Our previous study has demonstrated that the expression of *Δ113p53* is activated after heart injury and *Δ113p53*^+^ CMs undergo proliferation to contribute to zebrafish heart regeneration. Next, we used the *Δ113p53* promoter to generate the *Tg(Δ113p53:cmyc)* zebrafish transgenic line. Conditional overexpression of *cmyc* not only significantly promoted zebrafish CM proliferation and heart regeneration but also significantly enhanced *glut1* expression at the injury site. Inhibiting Glut1 diminished the increase in CM proliferation in *Tg(Δ113p53:cmyc)* injured hearts of zebrafish. Therefore, our results suggest that the activation of *cmyc* promotes heart regeneration through upregulating the expression of *glut1* to speed up glucose transportation.

## 1. Introduction

Cardiovascular diseases are the leading cause of human death around the world [1]. One of the important reasons for this is due to the limited regeneration capacity of adult mammalian hearts. Mice only retain the heart regeneration capacity before the first week after birth [2]. Unlike mammals, zebrafish can fully regenerate lost myocardium 60 days after injury through CM dedifferentiation and proliferation [3,4]. Thus, finding ways to promote CM proliferation provides a promising path to cure cardiovascular diseases.

To sustain repeated contractions, the heart needs an efficient and stable source of ATP production. The main source of ATP in the heart is mitochondrial oxidative phosphorylation of carbohydrates and lipids. During heart development, the amount of ATP produced by different metabolic pathways changes dramatically [5]. Before birth, mammalian fetuses are in a relatively hypoxic environment. The activities of the tricarboxylic acid cycle and electron transport chain are low, leading to weak oxidative phosphorylation ability. Therefore, glucose is the main carbon source and anaerobic glycolysis is the main source of energy production [6,7]. Shortly after birth, the heart metabolism rapidly shifts to fatty acid oxidation due to increased energy requirements. In 1-day-old rabbit hearts, glycolysis occupies about 50% of the total ATP production. However, this proportion is changed at 7 days after birth, with glycolysis accounting for only 5% and fatty acid oxidation rising to 39% [8], which coincides with the mammalian heart regeneration capacity window.

During zebrafish heart regeneration, single-cell sequencing reveals that there is a metabolic reprogramming process from oxidative phosphorylation to glycolysis in proliferating CMs [9]. Reducing fatty acid oxidation in neonatal mice promotes CM proliferation and cardiac regeneration and prolongs the postnatal cardiomyocyte proliferative window [10]. On the contrary, inhibition of the glycolytic pathway significantly represses proliferation and dedifferentiation of zebrafish CMs. For instance, depletion of *pyruvate kinase muscle isoenzyme a2* (*pkma2*), an orthologue of mammalian *Pkm2*, attenuates zebrafish CM proliferation and cardiac regeneration [11], whereas the conditional knockout of *pyruvate dehydrogenase kinase4* (*PDK4*), a negative regulator of Pyruvate Dehydrogenase (PDH), in CMs, results in an increase in glucose oxidation and CM proliferation in adult mice [12]. In the failing human heart, the expression of the *glucose transporter1 (Glut1)* is drastically downregulated [13] and overexpression of *Glut1* can prevent the development of heart failure in adult mice [14]. Cardiac-specific overexpression of *Glut1* also enhances glucose metabolism and promotes heart regeneration in neonatal mice [15]. However, it is still not fully understood how genes responsible for glucose transportation are regulated in heart regeneration.

*cMyc* is a well-known oncogene that can activate the expression of many glycolytic genes [16]. Overexpression of *Oct4*, *Sox2*, *Klf4* and *cMyc* (OSKM) in mouse hearts can induce adult cardiomyocytes to dedifferentiate and proliferate and extend the heart regeneration capacity window [17]. However, whether *cMyc* plays a role in metabolic shifts during heart regeneration is not clear. Our previous study revealed that the expression of *p53* isoform *Δ113p53* was activated in CMs at the resection site of zebrafish heart to promote CM proliferation. Cell lineage tracing showed that *Δ113p53*-positive CMs underwent cell proliferation to contribute to myocardial regeneration, which suggests that *Δ113p53*-positive CMs are proliferating cells [18]. Here, we observed that both Glut1 and cMyc were activated at the injury site of zebrafish heart. To investigate whether *cmyc* plays a role in *glut1* expression and heart regeneration, we used the *Δ113p53* promoter to generate *Tg(Δ113p53:cmyc)* transgenic zebrafish. The results showed that the *Tg(Δ113p53:cmyc)* transgene promotes zebrafish heart regeneration through the upregulation of *glut1*.

## 2. Results

### 2.1. Glut1 Expression and Glucose Uptake Are Upregulated around Injury Site of Zebrafish Hearts

**Figure 1:**
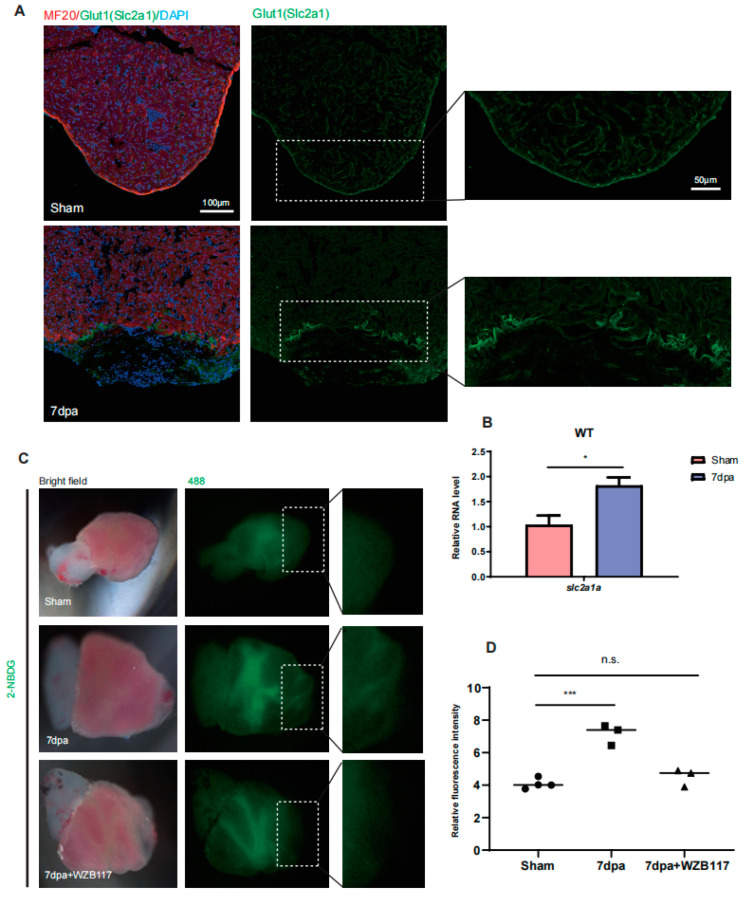
Glut1 expression and glucose uptake are upregulated around injury area of zebrafish hearts. (**A**) Immunostaining of MF20 (in red) and Glut1 (in green) in sham and injured hearts at 7 dpa. The nuclei were stained with DAPI (in blue). Framed areas were magnified in right panels. *n*: 5–6 hearts/sample. Scale bar: 100 μm or 50 μm as indicated. The experiment was repeated two times. (**B**) qRT-PCR was performed to examine the expression of *slc2a1a* in sham and injury (7 dpa) hearts. (**C**,**D**) Ex vivo glucose uptake assay. The ventricles were isolated from sham and injury hearts at 7 dpa. The isolated ventricles were incubated in 2-NBDG (a fluorescent derivative of glucose) solution for 1h, and treated with or without WZB117, a Glut1 inhibitor. The treated ventricles were captured in bright field (left panels) or 488 Channel (right panels) with a fluorescence microscope (**C**). The jelly-like tissues observed in the bright field images were injury areas. Framed areas were injury sties and were magnified in the right panels. The relative fluorescence intensity of the heart apex was analyzed with Image J (**D**). Each dot represents an individual heart (round dot, Sham group; frame dot, 7dpa group; triangle dot, 7dpa with WZB117 treatment group). *n*: 3–4 hearts/sample. The experiment was repeated three times. Statistical analysis was performed by Student’s two-tailed unpaired *t* test in GraphPad Prism 8. The *p* values were represented by n.s. and asterisks. n.s., *p* > 0.05; *, *p* < 0.05; ***, *p* < 0.001.

There are five different Glut (*Slc2a*) homologs: two forms of *slc2a1* (*slc2a1a* and *slc2a1b*), one form of *slc2a2* and two forms of *slc2a3* (*slc2a3a* and *slc2a3b*) in the zebrafish genome, corresponding to the mammalian *Slc2a1* (*Glut1*), *Slc2a2* (*Glut2*) and *Slc2a3* (*Glut3*), respectively. However, no homologs of mammalian *Slc2a4* (*Glut4*) are identified in zebrafish [19]. Transcriptomic analysis in a previous study showed that the transcription of *slc2a1a* (FPKM: 24) exhibits the highest level in zebrafish hearts among the five *glut* genes (*slc2a1b*: undetected; *slc2a2*: 0.07; *slc2a3a*: 0.17; *slc2a3b*: undetected), indicating that *slc2a1a* contributes the most to glucose transportation in zebrafish hearts [20]. Therefore, to investigate how glucose transportation is regulated in zebrafish heart regeneration, we used an antibody to exam Glut1 expression in injured hearts. The results showed that Glut1 was ubiquitously expressed in the ventricle of the sham heart and obviously upregulated in the injury site at 7 days post amputation (dpa) (Figure 1A). Quantitative Real-Time PCR (qRT-PCR) showed that the mRNA level of *slc2a1a* was also significantly increased after injury at 7 dpa (Figure 1B).

To study if the upregulation of Glut1 contributes to the uptake of glucose in the injury area, we isolated the ventricles of sham and injury hearts at 7 dpa. The isolated ventricles were cultured with 2-NBDG, a fluorescent derivative of glucose, for 1 h and treated with WZB117, a Glut1 inhibitor [21]. As the labeled glucose is a small molecule that will diffuse after tissue fixation and without Masson staining or other immunostaining, the injury area cannot be correctly identified in the cryosections. Therefore, it is technically impossible to use cryosections to evaluate the labeled glucose fluorescence intensity in the injury area. However, it is easily distinguished by its appearance (jelly-like tissue) in live injury hearts in the bright field pictures. Thus, we performed the assay of glucose uptake with live hearts. As expected, the fluorescence signal was observed in the chambers of either sham hearts or injury hearts with or without WZB117 treatment. Interestingly, the fluorescence intensity was significantly increased in the apex (around resection site) of the injury hearts compared to the apex of the sham hearts. The increase in the fluorescence intensity in the apex of the injury hearts was blocked by the treatment of WZB117 (Figure 1C,D). The results demonstrate that the expression of Glut1 is upregulated to promote the uptake of glucose in CMs around the injury site.

### 2.2. Glut1 Is Required for Zebrafish Heart Regeneration

**Figure 2:**
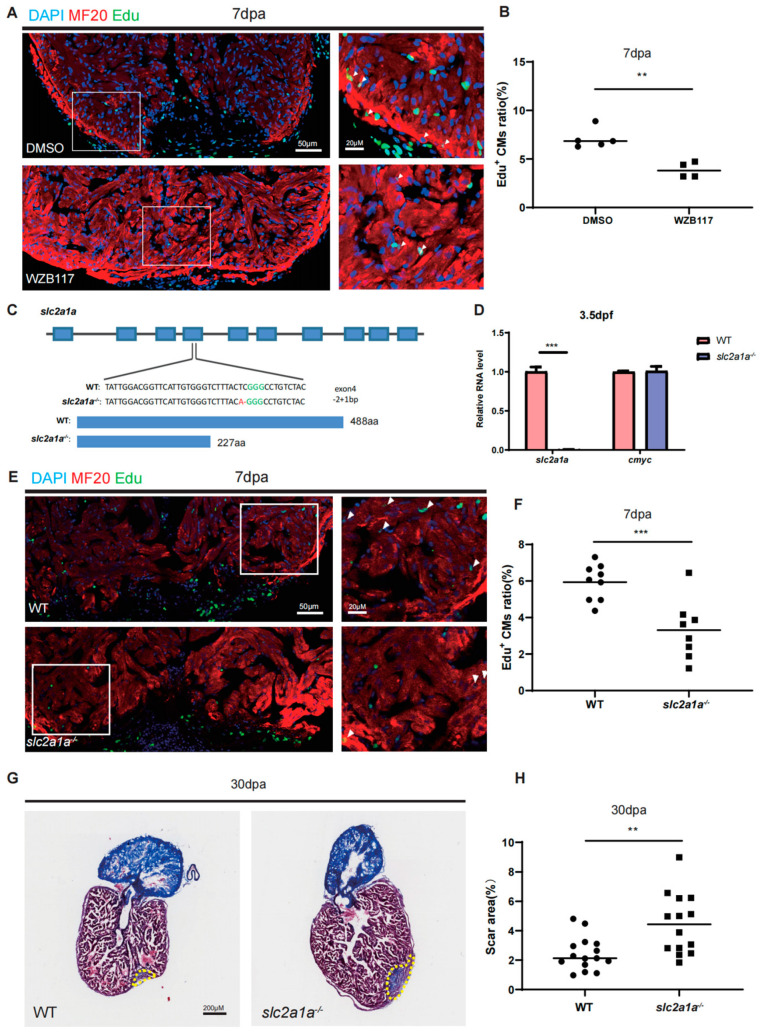
Depletion of Glut1 impairs zebrafish heart regeneration. (**A**) Cryosections of Edu-labelled (in green) zebrafish injury hearts with different treatments at 7 dpa were immunostained by anti-MF20 (in red). The nuclei were stained with DAPI (in blue). The zebrafish with heart resections were intraperitoneally injected with DMSO (the injection control) or WZB117. Framed areas were magnified in the right panels. Scale bar: 50 μm or 20 μm as indicated. (**B**) Statistical analyses of EDU^+^ CMs in A. The EDU^+^ CMs were presented as the percentage of the total MF20^+^ cells at the injury sites. Each dot represents an individual heart (round dot, DMSO injection group; frame dot, WZB117 injection group). *n*: 4–5 hearts/sample. The experiment was repeated three times. (**C**) Diagram showing the gRNA targeting site and two-bp deletion and one-bp insertion in the exon 4 of the *slc2a1a* mutant, which results in a premature stop codon (PTC) at 227 aa (the GGG in green is the PAM sequence). (**D**) qRT-PCR was performed to examine the expression of *slc2a1a* and *cmyc* in WT and *slc2a1a^−/−^* mutant embryos at 3.5 dpf. (**E**) Immunostaining of MF20 (in red) and EDU incorporation assay (in green) of WT and *slc2a1a^−/−^* injury hearts at 7 dpa. Framed areas were magnified in the right panels. Scale bar: 50 μm or 20 μm as indicated. (**F**) Statistical analyses of EDU^+^ CMs in C. The EDU^+^ CMs were presented as the percentage of the total MF20^+^ cells at the injury sites. Each dot represents an individual heart (round dot, WT group; frame dot, *slc2a1a^−/−^* group). *n*: 8–9 hearts/sample. The experiment was repeated two times. (**G**) Fibrin clot stained with Masson’s trichrome on the cryosections of WT and *slc2a1a^−/−^* injury hearts at 30 dpa. Yellow dotted lines indicate the approximate injury area. Scale bar: 200 μm. (**H**) Statistical analyses of scar areas in (**E**). Average injury area with fibrin clots on heart sections was presented as the percentage of the total ventricular area. Each dot represents an individual heart (round dot, WT group; frame dot, *slc2a1a^−/−^* group). *n*: 14–15 hearts/sample. Statistical analysis was performed by Student’s two-tailed unpaired *t* test in GraphPad Prism 8. The *p* values were represented by n.s. and asterisks. **, *p* < 0.01; ***, *p* < 0.001.

To investigate the function of Glut1 in zebrafish heart regeneration, we intraperitoneally co-injected the Glut1 inhibitor (WZB117) and EdU into WT zebrafish during 4–6 dpa. The animals were sacrificed at 7 dpa for the EdU incorporation assay. The results showed that the percentage of EDU^+^ CMs around the injury site at 7 dpa was significantly decreased in WZB117-injected hearts (3.89%) compared to that in DMSO-injected control hearts (7.08%) (Figure 2A,B).

To confirm the function of Glut1, we generated a zebrafish *slc2a1a* (*glut1*) mutant with 2 bp deletion and 1 bp insertion, using CRISPR/Cas9, resulting in a premature termination codon (PTC) at 227 aa (Figure 2C). The F0 fish was crossed with WT to screen the F1 single heterozygous allele. Then F1 mutant fish were inbred to generate F2 *slc2a1a^−/−^* homozygous mutant fish. qRT-PCR showed that the transcripts of *slc2a1a* were significantly decreased in the *slc2a1a^−/−^* mutant embryos at 3.5 days post fertilization (dpf), suggesting that the mutant mRNA with a PTC was degraded (Figure 2D). Knockout of *slc2a1a* did not have much effect on the transcription of *cmyc* (Figure 2D). A previous study found that knockdown of glut1 in zebrafish embryos resulted in sinus venosus defects [22]. However, the *slc2a1a^−^*^/*−*^ zebrafish mutants developed relatively normal and fertile and did not display obvious cardiac defects compared to WT. The possible reason for the phenotypic discrepancy between *slc2a1a* morphants and mutants may be due to the genetic compensation response. Then, we examined heart regeneration in *slc2a1a^−^*^/*−*^ mutants by apex resection. Similar to WZB117-injected hearts, the percentage of EDU^+^ CMs at 7 dpa was significantly decreased in *slc2a1a*^−/−^ zebrafish mutant hearts (3.31%) compared to that in WT hearts (5.94%) (Figure 2E,F). The Masson trichrome staining also showed that the scar area at 30 dpa was significantly larger in the *slc2a1a*^−/−^ mutant hearts (4.45%) than that in WT hearts (2.44%) (Figure 2G,H). The results demonstrate that the depletion of *slc2a1a* impairs zebrafish heart regeneration.

### 2.3. The Expression of cMyc Is Activated around the Injury Site of Zebrafish Hearts, Which Is Excessively Upregulated in Tg(Δ113p53:cmyc) Transgenic Zebrafish Injury Hearts

**Figure 3:**
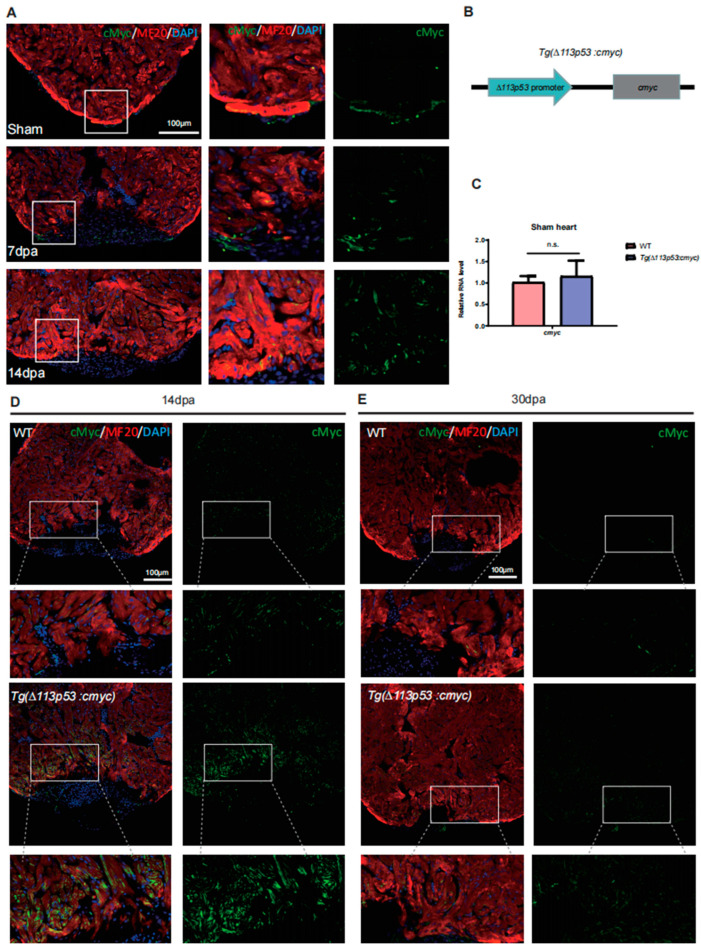
The expression of cMyc is activated around the injury site of zebrafish hearts, which is excessively upregulated in *Tg(Δ113p53:cmyc)* transgenic zebrafish injury hearts. (**A**) Immunostaining of MF20 (in red) and cMyc (in green) in WT sham and injury hearts at 7 and 14 dpa. Framed areas were magnified in right panels. *n*: 8–11 hearts/sample. Scale bar: 100 μm. (**B**) Diagram showing the construction of *Tg(∆113p53:cmyc)* transgene. (**C**) qRT-PCR was performed to exam the expression of *cmyc* in WT and *Tg(∆113p53:cmyc)* sham hearts. (**D**,**E**) Immunostaining of MF20 (in red) and cMyc (in green) of WT and *Tg(∆113p53:cmyc)* hearts at 14 dpa (**D**) and 30 dpa (**E**). Framed areas were magnified in lower panels. *n*: 10–12 hearts/sample. Scale bar: 100 μm. Statistical analysis was performed by Student’s two-tailed unpaired *t* test in GraphPad Prism 8. The *p* values were represented by n.s. and asterisks. n.s., *p* > 0.05.

Previous studies have demonstrated that *cMyc* can regulate the expression of many metabolic genes [16]. Overexpression of OSKM transcription factors stimulates CMs in adult mouse hearts to reenter cell cycle [17]. To explore if *cMyc* plays a role in regulation of metabolic shifts during zebrafish heart regeneration, we used an antibody to exam cMyc expression in injury hearts. The results showed that the expression of cMyc was detected only in the epicardium of sham ventricles and was obviously upregulated around the injury site at 7 and 14 dpa (Figure 3A). The results demonstrate that heart injury can trigger the activation of cMyc.

Depletion of zebrafish *cmyc* with morpholino or genetic knockout results in various abnormal developments in zebrafish embryogenesis, suggesting *cmyc* knockout zebrafish mutants are not able to grow into the adult stage [23]. To explore whether the activation of cMyc promotes glut1 expression and heart regeneration, we conditionally expressed *cmyc* in proliferating CMs, which can prevent the side effects of overexpression of *cmyc*. Studies from our lab have revealed that *Δ113p53* is an N-terminal truncated isoform of p53 and functions to antagonize p53-mediated apoptosis and promote DNA double-strand breaks [24,25,26,27]. Our further study has demonstrated that the expression of *Δ113p53* is induced in CMs around the injury area of zebrafish hearts and *Δ113p53*^+^ CMs undergo DNA synthesis to contribute to zebrafish heart regeneration [18]. The results indicate that *Δ113p53*^+^ cells are proliferating CMs. To conditionally express *cmyc* in proliferating CMs, we used the *Δ113p53* promoter to create *Tg(Δ113p53:cmyc)* transgenic zebrafish (Figure 3B). No obvious phenotypes were observed in *Tg(∆113p53:cmyc)* zebrafish. Similar to the WT sham hearts, the expression of cMyc remained at a very low level in *Tg(Δ113p53:cmyc)* zebrafish sham hearts (Figure 3C). Our previous research demonstrated that the expression of *Δ113p53* reached its highest level at 14 dpa and then gradually decreased [18]. Therefore, we detected the expression of cMyc at 14 and 30 dpa. Immunostaining showed that the expression of cMyc was obviously upregulated around the injury site in *Tg(Δ113p53:cmyc)* hearts at 14 dpa compared to that in WT injury hearts (Figure 3D). The expression of cMyc was decreased at 30 dpa in *Tg(Δ113p53:cmyc)* injury hearts compared to that in *Tg(Δ113p53:cmyc)* hearts at 14 dpa (Figure 3E). The results showed that the expression of cMyc in *Tg(Δ113p53:cmyc)* hearts mimicked the induction of *∆113p53* in response to heart injury.

**Figure 4:**
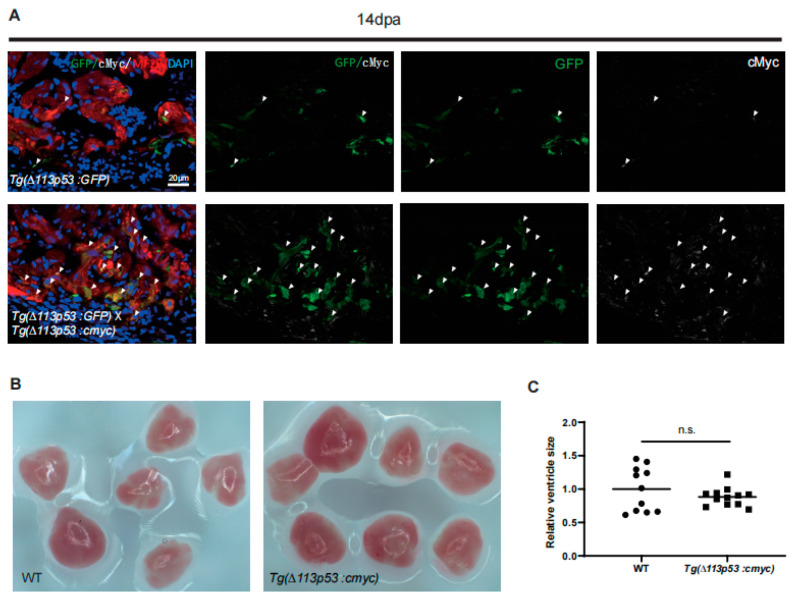
cMyc is conditionally overexpressed in *∆113p53*^+^ CMs in *Tg(∆113p53:cmyc)* zebrafish hearts. (**A**) Immunostaining of MF20 (in red), GFP (in green) and cMyc (in white) in *Tg(∆113p53:GFP)* single and *Tg(Δ113p53:cmyc);(TgΔ113p53:GFP)* double transgenic zebrafish hearts at 14 dpa. Scale bar: 20 μm. *n*: 11–12 hearts/sample. (**B**,**C**) Representative pictures of WT and *Tg(∆113p53:cmyc)* adult zebrafish hearts (**B**). The statistical analysis of the average ventricle sizes of *Tg(∆113p53:cmyc)* and WT adult zebrafish is shown in (**C**). Each dot represents an individual heart (round dot, WT group; frame dot, *Tg(∆113p53:cmyc)* group). *n*: 11–12 hearts/sample. Statistical analysis was performed by Student’s two-tailed unpaired *t* test in GraphPad Prism 8. The *p* values were represented by n.s. and asterisks. n.s., *p* > 0.05.

In our previous studies, we generated a *tg(Δ113p53:GFP)* transgenic line, in which the expression of GFP genially reflects the transcription of endogenous *Δ113p53* [25]. To confirm that *cmyc* is indeed overexpressed in *∆113p53*^+^ CMs, we crossed *Tg(Δ113p53:cmyc)* with *Tg(Δ113p53:GFP)* to generate the *Tg(Δ113p53:cmyc)*;(*TgΔ113p53:GFP)* double transgenic line. Immunostaining showed that there were some cMyc-positive CMs around the injury area of the heart in the *T*g(*Δ113p53:GFP)* single transgenic line, and some of cMyc signals were co-localized with GFP signals, suggesting that both cMyc and *Δ113p53* were activated in proliferating CMs in response to heart injury. In *Tg(Δ113p53:cmyc)*;(*TgΔ113p53:GFP)* double transgenic animals, cMyc-positive CMs were drastically increased in the injury area and most of the cMyc signals were overlapped with GFP signals (Figure 4A). The results demonstrated that the expression of cMyc was excessively upregulated only in *∆113p53*^+^ CMs of *Tg(Δ113p53:cmyc)* injury hearts but not in normal conditions. In addition, the heart of *Tg(Δ113p53:cmyc)* grew at normal size and displayed no obvious difference from WT hearts (Figure 4B,C).

Taken together, the expression of cMyc is activated around the injury site of zebrafish hearts and is over-upregulated only in proliferating CMs of *Tg(Δ113p53:cmyc)* injury hearts.

### 2.4. Tg(Δ113p53:cmyc) Transgene Promotes CM Proliferation and Heart Regeneration

**Figure 5:**
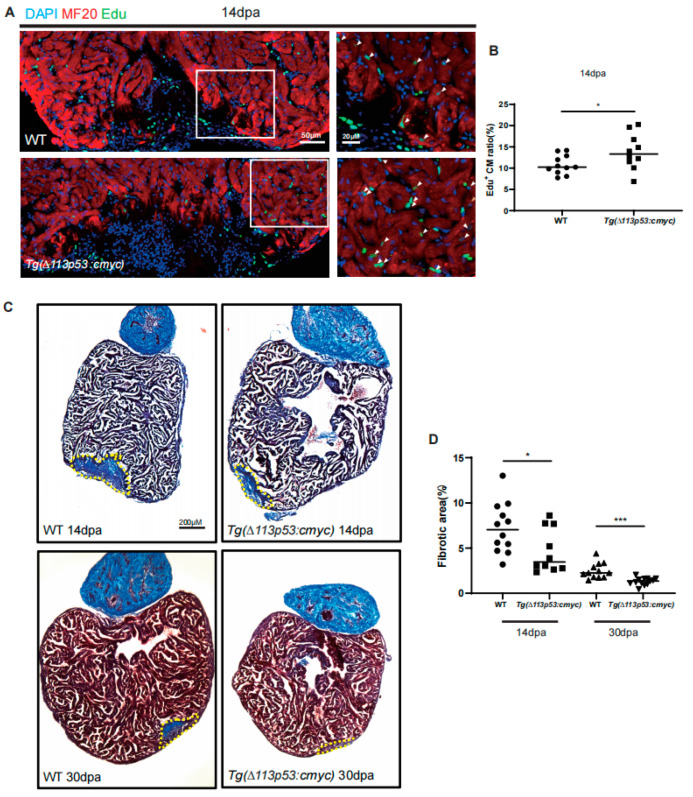
Conditional overexpression of *cmyc* in *∆113p53^+^* cells promotes zebrafish CM proliferation and heart regeneration. (**A**) Immunostaining of MF20 (in red) and EDU incorporation assay (in green) of WT and *Tg(∆113p53:cmyc)* injury hearts at 14 dpa. Framed areas were magnified in right panels. Scale bar: 50 μm or 20 μm as indicated. (**B**) Statistical analyses of EDU^+^ CMs in A. The EDU^+^ CMs were presented as the percentage of the total MF20^+^ cells at the injury sites. Each dot represents an individual heart (round dot, WT group; frame dot, *Tg(∆113p53:cmyc)* group). *n*: 10–11 hearts/sample. The experiment was repeated three times. (**C**) Fibrin clot stained with Masson’s trichrome on the cryosections of WT and *Tg(∆113p53:cmyc)* injury hearts at 14 and 30 dpa. Yellow dotted lines indicate the approximate injury area. Scale bar: 200 μm. (**D**) Statistical analyses of scar areas in (**C**). Average injury area with fibrin clots on heart sections was presented as the percentage of the total ventricular area. Each dot represents an individual heart (round dot, WT at 14 dpa group; frame dot, *Tg(∆113p53:cmyc)* at 14 dpa group; positive triangle dot, WT at 30 dpa group; inverted triangle dot, *Tg(∆113p53:cmyc)* at 30 dpa group). *n*: 10–12 hearts/sample. Statistical analysis was performed by Student’s two-tailed unpaired *t* test in GraphPad Prism 8. The *p* values were represented by n.s. and asterisks. *, *p* < 0.05; ***, *p* < 0.001.

Next, we examined the function of the *T*g(*Δ113p53:cmyc)* transgene on heart regeneration. The percentage of EDU^+^ CMs at 14 dpa was significantly increased in *T*g(*Δ113p53:cmyc)* transgenic zebrafish hearts (13.8%) compared to that in WT hearts (10.7%) (Figure 5A,B). The Masson trichrome staining also showed that the scar area at either 14 or 30 dpa was significantly smaller in the *Tg(Δ113p53:cmyc)* transgenic zebrafish hearts (4.73% and 1.31%) than the respective control in WT hearts (7.22% and 2.48%) (Figure 5C,D). The results demonstrate that overexpression of cmyc in proliferating CMs accelerates the zebrafish heart regeneration process.

### 2.5. Tg(Δ113p53:cmyc) Transgene Promotes Zebrafish CM Proliferation via Upregulation of Glut1 Expression

**Figure 6:**
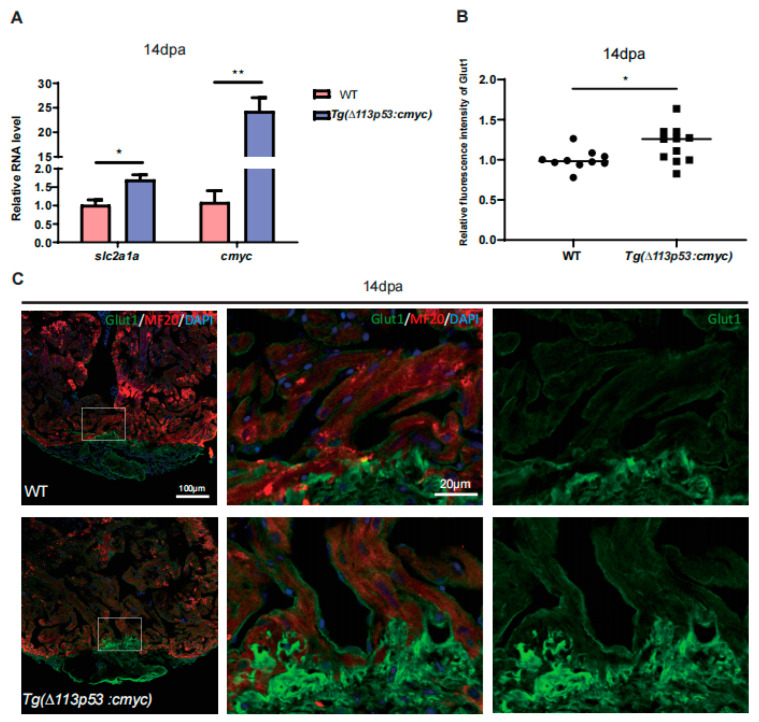
Conditional overexpression of *cmyc* in *∆113p53^+^* cells activates Glut1 expression. (**A**) qRT-PCR was performed to examine the expression of *slc2a1a* and *cmyc* in WT and *Tg(∆113p53:cmyc)* injury hearts at 14 dpa. (**B**,**C**) Immunostaining of MF20 (in red) and Glut1 (in green) in WT and *Tg(∆113p53:cmyc)* injury hearts at 14 dpa (**C**) and statistical analyses of Glut1 relative fluorescence intensity around the injury site in (**C**). (**B**). Framed areas were magnified in right panels. Each dot represents an individual heart (round dot, WT group; frame dot, *Tg(∆113p53:cmyc)* group). *n*: 10–11 hearts/sample. Scale bar: 100 μm or 20 μm as indicated. The experiment was repeated two times. Statistical analysis was performed by Student’s two-tailed unpaired *t* test in GraphPad Prism 8. The *p* values were represented by n.s. and asterisks. *, *p* < 0.05; **, *p* < 0.01.

To investigate whether *cmyc* plays a role in the regulation of Glut1 expression during heart regeneration, we performed qRT-PCR to detect *slc2a1a* (*glut1*) mRNA level in *T*g(*Δ113p53:cmyc)* injury hearts at 14 dpa. The results showed that the expression of both *cmyc* and *slc2a1a* mRNAs was significantly increased in *T*g(*Δ113p53:cmyc)* injury hearts, compared to those in WT injury hearts (Figure 6A). Immunostaining also showed that the protein level of Glut1 was also significantly higher around the injury site in *T*g(*Δ113p53:cmyc)* injury hearts at 14 dpa than that in WT injury hearts (Figure 6B,C). Therefore, cMyc can indeed activate Glut1 expression in the injury hearts.

**Figure 7:**
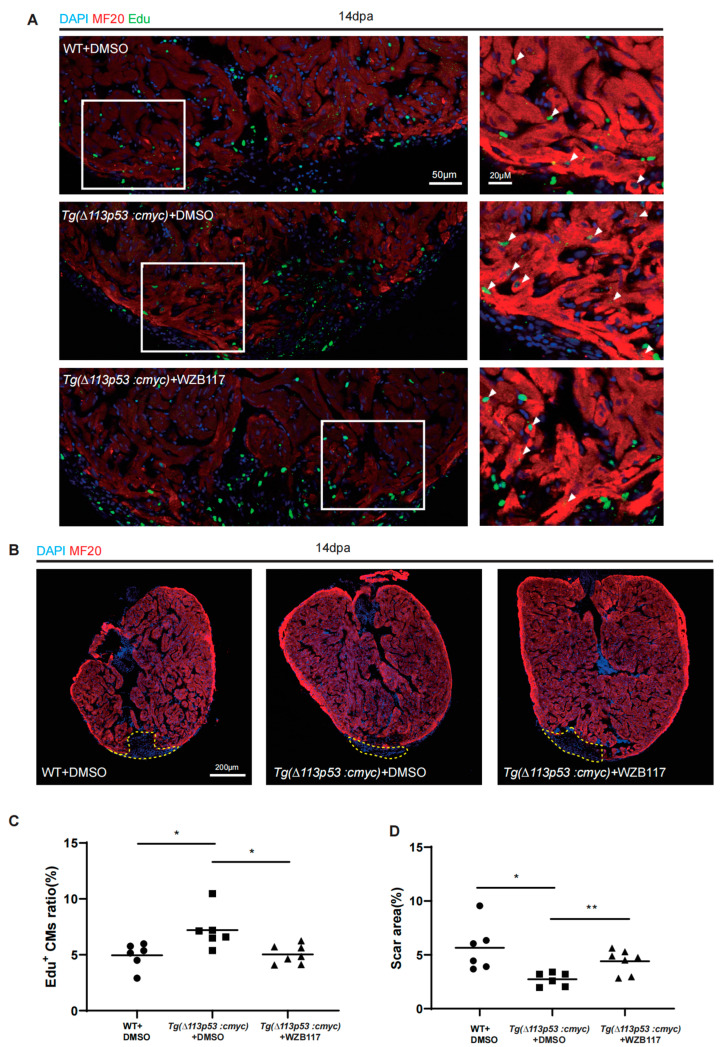
Conditional overexpression of *cmyc* in *∆113p53^+^* cells promotes heart regeneration dependent on Glut1. (**A**) Immunostaining of MF20 (in red) and EDU incorporation assay (in green) of injury hearts at 14 dpa in WT and *Tg(∆113p53:cmyc)* zebrafish intraperitoneally injected with DMSO (the injection control) or WZB117. The nuclei were stained with DAPI (in blue). Framed areas were magnified in right panels. Scale bar: 50 μm or 20 μm as indicated. (**B**) Immunostaining of MF20 (in red) of injury hearts at 14 dpa in WT and *Tg(∆113p53:cmyc)* zebrafish intraperitoneally injected with DMSO (the injection control) or WZB117. The nuclei were stained with DAPI (in blue). Scale bar: 200 μm. (**C**) Statistical analyses of EDU^+^ CMs in (**A**). The EDU^+^ CMs were presented as the percentage of the total MF20^+^ cells at the injury sites. Each dot represents an individual heart (round dot, WT with DMSO injection group; frame dot, *Tg(∆113p53:cmyc)* with DMSO injection group; triangle dot, *Tg(∆113p53:cmyc)* with WZB117 injection group). *n*: 6–7 hearts/sample. The experiment was repeated two times. (**D**) Statistical analyses of scar areas in (**B**). Average injury area without MF20 signals on heart sections was presented as the percentage of the total ventricular area. Each dot represents an individual heart (round dot, WT with DMSO injection group; frame dot, *Tg(∆113p53:cmyc)* with DMSO injection group; triangle dot, *Tg(∆113p53:cmyc)* with WZB117 injection group). *n*: 6–7 hearts/sample. The experiment was repeated two times. Statistical analysis was performed by Student’s two-tailed unpaired *t* test in GraphPad Prism 8. The *p* values were represented by n.s. and asterisks. *, *p* < 0.05; **, *p* < 0.01.

To evaluate the facilitation of the *T*g(*Δ113p53:cmyc)* transgene in heart regeneration in the context of *glut1* expression, we intraperitoneally injected the Glut1 inhibitor WZB117 into *T*g(*Δ113p53:cmyc)* during 11–13 dpa. Similar to the above result, the percentage of EDU^+^ CMs at 14 dpa was significantly increased in *T*g(*Δ113p53:cmyc)* transgenic zebrafish hearts compared to that in WT hearts. However, the increase in EDU^+^ CMs at 14 dpa in *T*g(*Δ113p53:cmyc)* transgenic zebrafish hearts was abolished by the injection of WZB117 (Figure 7A,C). The immunostaining of MF20 also showed that the scar area at 14 dpa was significantly decreased in *T*g(*Δ113p53:cmyc)* transgenic zebrafish injury hearts compared to that in WT injury hearts, while the injection of WZB117 resulted in a significant increase in scar area in *T*g(*Δ113p53:cmyc)* transgenic zebrafish injury hearts (Figure 7B,D).

Thus, the results demonstrate that cMyc promotes the expression of glut1 to accelerate glucose transportation and heart regeneration.

## 3. Discussion

In this report, we found that Glut1 expression and glucose uptake were upregulated around the injury area of zebrafish hearts. Inhibiting Glut1 with a Glut1 inhibitor WZB117 not only abolished the increase in glucose uptake in the injury site but also impaired zebrafish heart regeneration. The facilitation of Glut1 in heart regeneration was confirmed by a genetic mutant of *slc2a1a*. Therefore, the results have demonstrated that upregulation of Glut1 plays an important role in metabolic remodeling and is essential for zebrafish heart regeneration.

Developmental change and injury response of the heart lead to a metabolic shift to glycolysis for energy production [28]. Cancer cells have a higher capacity of proliferation and there exists a Warburg effect. That is, cancer cells take up glucose at higher rates compared with normal tissues [29]. For sharing a similar metabolism in tumor tissues and heart failure, there exists a correlation between tumor formation and regeneration [30]. It is generally agreed that cancer cells display enhanced glycolysis [31]. Additionally, oncogene cMyc had been reported to activate many glycolysis pathway genes [32]. So, we wonder whether cMyc plays a role in regulating glucose uptake to participate in heart regeneration.

We observed that the expression of cMyc was also upregulated around the injury area of zebrafish hearts. To investigate if the activation of cMyc regulates the expression of Glut1, we used the *Δ113p53* promoter to conditionally overexpress cmyc in proliferating CMs in response to heart injury. The expression of cMyc was upregulated only around the injury area in *T*g(*Δ113p53:cmyc)* transgenic zebrafish hearts, which reflected the induction of *Δ113p53* during zebrafish heart regeneration. Conditional overexpression of *cmyc* in proliferating CMs did not result in abnormal heart growth and tumor formation. However, the percentage of proliferated CMs during heart regeneration at 14 dpa was significantly increased, whereas the scar area was significantly decreased at 30 dpa in *T*g(*Δ113p53:cmyc)* transgenic fish. Thus, conditional overexpression of cMyc in proliferated CMs promotes heart regeneration without obvious side effects. We also noticed that cMyc expression was not limited to the injury-adjacent CMs of zebrafish hearts at 7 dpa. Our previous study showed that the expression of *Tg(Δ113p53:GFP)* is induced in CMs around the injury area of zebrafish hearts and *Δ113p53^+^* CMs undergo DNA synthesis to contribute to zebrafish heart regeneration. However, we cannot exclude that *Δ113p53* might also be expressed in non-CM cells [18]. Thus, it is also possible that cMyc functions in other cell types to promote heart regeneration.

Finally, we used *T*g(*Δ113p53:cmyc)* transgenic fish to investigate the relationship between *cmyc* and *glut1* in heart regeneration. We found that the increases in both mRNA and the protein of Glut1 around the injury site of *Tg(Δ113p53:cmyc)* transgenic fish hearts were moderate but significant. One of reasons for the moderate increase of Glut1 in the transgenic fish injury heart is that the expression of Glut1 has already been induced by heart injury in WT fish, which may mask the function of the transgene. Inhibiting Glut1 significantly attenuated the increase in CM proliferation and heart regeneration in *T*g(*Δ113p53:cmyc)* transgenic fish.

In summary, in response to heart injury, the expression of cMy*c* is activated to promote *glut1* expression. Glut1 enhances glucose uptake to facilitate CM proliferation. However, how the expression of cMyc is upregulated in zebrafish heart regeneration is required for further investigation.

## 4. Materials and Methods

### 4.1. Zebrafish Lines

Zebrafish were raised and maintained in standard zebrafish units at Zhejiang University as described previously [33]. Zebrafish AB stain was used to generate the *slc2a1a^−^*^/*−*^ mutant and *Tg(Δ113p53:cmyc)* transgenic fish. The *Tg(Δ113p53:GFP)* transgenic line was generated in our previous studies. A 3.6-kb fragment of the *Δ113p53* promoter as described in our previous study was used to create the *Tg(Δ113p53:cmyc)* transgenic line on the AB genetic background through Tol2-based transgenesis.

The gRNA sequences were listed as follows: slc2a1a forward gRNA primer (ATAATACGACTCACTATAGTTCATTGTGGGTCTTTACTCGTTTTAGAGCTAGAAATAGC); slc2a1a reverse gRNA primer (AGCACCGACTCGGTGCCACT). Sequences with bold fonts (GTTCATTGTGGGTCTTTACTC) in the forward gRNA primer are from slc2a1a. The upstream sequence is the T7 promoter. The downstream sequence and the reverse gRNA primer are the gRNA scaffold.

### 4.2. Zebrafish Heart Apex Resection

Ventricular surgery was performed on 6- to 12-month-old zebrafish according to previously described procedures. Briefly, zebrafish were anaesthetized with 0.02% Tricaine and then subjected to ~15% ventricular amputation at the apex with scissors. Sham procedures excluded apex resection.

### 4.3. Quantitative Real-Time Reverse Transcriptional PCR (qRT-PCR)

Hearts were freshly isolated from anaesthetized zebrafish subjected to sham surgery or resection at different time points. The outflow tracts and atriums were removed from the isolated hearts. Total RNA was extracted from approximately 4 to 6 isolated ventricles from each group using TRIZOL reagent (Invitrogen,15596026). For the experiments in zebrafish embryos, total RNA was isolated from 40 to 60 embryos in each group. About 1 μg isolated RNA was treated with DNaseI (NEB, M0303S) prior to reverse transcription and purified through lithium chloride. First-strand cDNA was synthesized using M-MLV Reverse Transcriptase (Invitrogen, C28025021). The reaction was performed using a CFX96^TM^ Real-Time System (Bio-Rad, America) with AceQ qPCR SYBR Green (Vazyme, Q111–02) according to the manufacturer’s instructions. Total RNA levels were normalized to the level of g*apdh*. Statistics were obtained from three repeats.

The primer sequences of the analyzed genes are listed as follows: slc2a1a qRT-PCR primer (TTGTGGGTCTTTACTCGGGC;ATGAGGAGGTATCGTGGGCT); cmyc qRT-PCR primer (GGATCTGAGCACCTCTGCAT;CGACTCTGAAGCATCCGTCT); gapdh qRT-PCR primer (TTCCAGTACGACTCCACCCA;TGACTCTCTTTGCACCACCC); β-actin qRT-PCR primer (CATTGGCAATGAGCGTTTC;TACTCCTGCTTGCTGATCCAC).

### 4.4. Drug Administration

For the zebrafish in vivo experiment, 15 μL of 100 μM WZB117 (GLPBIO, GC13381) was intraperitoneally injected in each fish at 4 to 6 dpa or 11 to 13 dpa. For the ex vivo experiment, isolated hearts were pre-treated in 40 μM WZB117 for 15 min and then incubated in 20 μM 2-NBDG (GLPBIO, GC10289, 20 μg/mL) and 40 μM WZB117 for 1 h. The treated dose of WZB117 was determined based on previous study [34].

### 4.5. Glucose Uptake Assay

After incubation in 2-NBDG, the isolated hearts were washed with PBS 3 times and photographed under a fluorescence microscope with a 488 channel. The relative fluorescence intensity of the apex in the images was analyzed by Image J.

### 4.6. EdU Incorporation Assay

For the EdU assays, 3 μL of 500 mM EdU (Invitrogen, A10044) was intraperitoneally injected into each zebrafish once daily from 4 to 6 dpa or 11 to 13 dpa. EdU staining was performed by Azide Alexa Fluor 647 (Invitrogen, A10277).

### 4.7. Masson Staining

Serial sections around the zebrafish heart injury area were collected for each individual heart. The average of the scar area was calculated from more than 10 hearts of each group. Masson staining and photographs were performed by Haoke Biotechnology CO (Hangzhou, China).

### 4.8. Immunostaining and Histological Methods

The cryosection was performed as previously described [35]. For immunostaining, the primary antibodies were anti-MF20 (DSHB, AB 2147781, 1:50), anti-Glut1 (ABclonal, A6982, 1:200), anti-cmyc (ABclonal, A1309, 1:200), anti-GFP (Abcam, ab13970). The second antibodiess were anti-mouse IgG H&L Alexa Fluor 488 (Abcam, ab150113), anti-Chicken IgY H&L Alexa Fluor 488 (Abcam, ab150169), Dylight 549-conjugated anti-rabbit IgG H&L (EarthOx, E032320), anti-Rabbit IgG H&L Alexa Fluor 647 (Abcam, ab150143), anti-mouse IgG H&L Alexa Fluor 647 (Abcam, ab150115). Nuclei were stained by DAPI (BYT, C1002).

### 4.9. Statistical Analysis

Sample sizes were designed based on routine genetic analysis in zebrafish. Unless stated otherwise, the experiments were randomized and investigators were not blinded to allocation during experiments. No data were excluded from the analyses. Unless stated otherwise, all parameters were tested using unpaired two-tailed Student’s *t* test. Significant *p*-values in all statistical analyses were obtained using GraphPad Software (Graphpad Prism 8.3.0). A *p*-value below 0.05 was considered to be statistically significant (*p* > 0.05, n.s. *p* < 0.05, * *p* < 0.01, ** *p* < 0.001, ***).

## 5. Conclusions

In summary, our study has revealed that cMyc is activated around the injury site of zebrafish hearts. The activation of cMyc upregulates the expression of glut1 to enhance glucose transportation in proliferated CMs, which is essential for heart regeneration. The *Δ113p53* promoter also provides a system to conditionally overexpress those important developmental genes in proliferating CMs to study their functions in heart regeneration without side effects.

## Data Availability

The raw data of this article will be made available by the authors, without undue reservation.
